# Two-Stage Channel Estimation for Semi-Passive RIS-Assisted Millimeter Wave Systems

**DOI:** 10.3390/s22155908

**Published:** 2022-08-07

**Authors:** Chengzuo Peng, Honggui Deng, Haoqi Xiao, Yuyan Qian, Wenjuan Zhang, Yinhao Zhang

**Affiliations:** School of Physics and Electronics, Central South University, Lushan South Road, Changsha 410083, China

**Keywords:** reconfigurable intelligent surface, semi-passive, channel estimation, multiple-input multiple-output

## Abstract

In a reconfigurable intelligent surface (RIS) assisted millimeter Wave (mmWave) communication system, the channel coefficient increases exponentially with the number of RIS elements which results in expensive pilot overhead. Most previous works have proposed some channel estimation algorithms for the estimation accuracy of cascaded channels, which have improved the estimation accuracy, but the pilot overhead is discouraging in the estimation process. To improve the channel estimation accuracy with reduced pilot overhead, we propose a two-stage channel estimation protocol by exploiting semi-passive elements and the coherent time difference of the channel, where the quasi-static channel between the base stations (BS) and RIS is estimated at the RIS, and the user (UE)-RIS time-varying channel is estimated at the BS. In the first stage, we formulate the BS-RIS channel estimation as a mathematical optimization problem by an iterative weighting method and then propose a gradient descent (GD)-based algorithm to solve it. In the second stage, we first transform the received the UE-RIS signal model into an equivalent parallel factor (PARAFAC) tensor model and estimate the UE-RIS channel by the least-squares (LS) algorithm. The simulation results show that the proposed method has better estimation accuracy than the LS, compression sensing (CS) and minimum mean square error (MMSE) methods with less pilot overhead, and the spectral efficiency is improved by at least 10.5% compared to the other three methods.

## 1. Introduction

The emerging reconfigurable intelligent surface (RIS) technology has been identified as a promising key technology for future 6G communications. RIS consists of a large number of low-cost passive reflective elements in a planar array, which enhance communication performance by subtly changing the direction of signal transmission [1,2,3,4]. Specifically, by digital reconfigurable/programmable meta-surface technology, each passive element appropriately adjusts the amplitude and phase of the incident signal to flexibly configure the wireless channel between the transmitter and receiver, and reduce fading impairment and interference problems in the wireless channel, thus enhancing communication performance. Existing research indicates that RIS can significantly improve communication rates [5,6], cancel interference, and expand network coverage, etc. However, the performance gain achieved by RIS relies on accurate channel state information (CSI). Consequently, accurate CSI estimation is critical for RIS-assisted communication systems.

However, the following two problems make acquiring CSI extremely difficult. Firstly, due to a large number of reflective elements of the RIS [7], the channel coefficients that need to be estimated subsequently increase, which leads to an expensive pilot overhead. Secondly, conventional RIS lacks active transmitters and receivers with signal processing capability, and it is difficult to estimate the exact the base station (BS)-RIS and RIS-user equipment (UE) channels separately, further complicating channel estimation. As a result, channel estimation is challenging for RIS-aided wireless communications.

There has been limited research on channel estimation for RIS-assisted communication systems to obtain accurate CSI. Most works are devoted to estimating cascaded channels [8,9,10,11,12,13,14,15]. The works [8,9] estimated cascaded channels by using the conventional least-squares (LS) algorithm, which was computationally simple, but it was sensitive to noise and does not measure with high accuracy in the case of few pilots. In [10], the least squares algorithm was improved by the proposed minimum mean square error (MMSE) channel estimator, which reduced the effect of noise on the channel estimation and improved the estimation accuracy. The work [11] described the channel estimation problem as an estimation error minimization problem and proposed a Lagrange multiplier and a pairwise ascending one to solve this problem, which improved the accuracy to some extent but increased the computational complexity. The work [12] proposed a three-stage channel estimation framework to improve the estimation accuracy by exploiting the channel consistency among UEs, but the pilot overhead still grows proportionally to the RIS elements. The work [13,14] proposed a sparse matrix decomposition and complementary channel estimation method based on sparse matrix decomposition to reduce the pilot overhead by exploiting channel sparsity. In addition, the work [15] proposed compressed sensing(CS) techniques to estimate channels for high-frequency millimeter wave(mmWave), exploiting the sparsity of mmWave to reduce the pilot overhead, but the computational complexity grows exponentially with the number of RIS elements.We summarize previous work in Table 1.

Despite the high channel estimation performance achieved in the above works, it is difficult to estimate the BS-RIS channel and the UE-RIS channel separately due to the passive nature of the RIS. In other words, if each RIS element can sense and reflect the signal, CSI can be easily estimated separately at the RIS. However, the method requires a large number of semi-passive elements with radio frequency (RF) chains, which results in higher hardware cost and power consumption. To tackle this problem, a small number of semi-passive components were proposed in [16,17,18] for receiving and processing the pilot signals in the RIS, and the BS-RIS channel and the UE-RIS channel were estimated separately based on the received pilot signals by the CS algorithm. However, the CS algorithm usually involves complex non-convex optimization problems, and it is difficult to obtain optimal solutions.

Based on the above discussion, we can observe that the problem of obtaining accurate CSI with low pilot overhead requires more research. The paper investigates the channel estimation problem for a semi-passive RIS-assisted mmWave multiple-input multiple-output (MIMO) system. We propose a two-stage channel estimation scheme to achieve high accuracy channel estimation with low pilot overhead. The main contributions of this paper are as follows:

- For the problem of expensive pilot overhead due to many channel coefficients, we introduce semi-passive elements to assist channel estimation and then propose a channel estimation protocol based on the coherence time difference between BS-RIS quasi-static channels and time-varying UE-RIS channels, in which the BS-RIS channels are estimated at long time scales using semi-passive elements and the BS estimates the UE-RIS channels at short time scales.The proposed channel estimation protocol is able to reduce the average pilot overhead at long time scales.
- To estimate BS-RIS channel, we propose an iterative re-weighting-based super-resolution algorithm to estimate the BS-RIS channel. We transform the BS-RIS channel estimation problem to the optimization problem of a new objective function by an iterative weighting method, which is the weighted summation of the sparsity and the data fitting error, Then we propose a gradient descent method to solve the objective function problem and update the weight parameters at each iteration to balance the sparsity with the data fitting error. During the iterative process, the estimated parameters move gradually to the neighborhood of the true value.Compared to traditional algorithms, the proposed algorithm is able to converge the estimates to near the true value and achieve accurate estimates.
- To estimate the time-varying channel of the UE-RIS, we propose a LS algorithm based on parallel factor(PARAFAC) decomposition to estimate the time-varying channel of the UE-RIS. We transform the received signal model into an equivalent PARAFAC tensor model, then obtain the the UE-RIS channel by LS algorithm.The proposed algorithm has higher robustness compared to the traditional algorithm by using PARAFAC decomposition, which avoids the problem of non-existence of matrix inverse.

The rest of this paper is presented as follows: Section 2 introduces the MIMO system model with the assistance of semi-passive RIS and the channel transmission protocol. In Section 3, we propose a channel estimation algorithm for the BS-RIS channel. In Section 4, we propose a channel estimation algorithm for the UE-RIS channel. The simulation results and the conclusion remarks are provided in Section 5 and  Section 6, respectively.

## 2. Channel Model and Channel Estimation Protocol

In this section, we present the channel model of the RIS-assisted mmWave communication system and the proposed channel estimation protocol.

### 2.1. Channel Model

**Figure 1:**
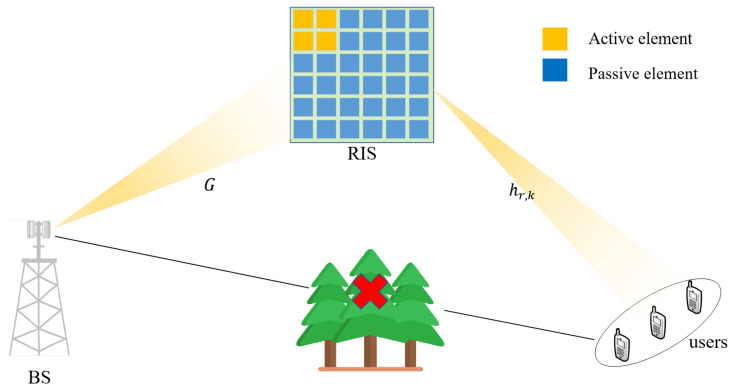
Semi-passive RIS-assisted mmWave massive MIMO system.

As shown in Figure 1, we consider a semi-passive RIS-assisted mmWave MIMO communication system, which consists of an *M* antenna base station, a semi-passive RIS, and *K* single-antenna users. The RIS consists of $N_{0}$ semi-passive elements and $N_{1}$ passive elements, while the semi-passive elements have an RF chain capable of receiving and processing signals when it is turned on, among $N_{0}+N_{1}=N$, $N_{0}<<N$. Due to the presence of semi-passive elements, the RIS has two modes: receive mode and reflect mode. In the receive mode, the semi-passive elements are used for channel estimation. In the reflection mode, the semi-passive elements and passive elements reflect the signal to the receiver. In this paper, we assume that the channel between BS and UE is blocked. Then there are two channels in this path, which are the BS-RIS channel $G\in C^{N\times M}$ and the UE-RIS channel $h_{r,k}\in C^{N\times K}$. In addition, since the adjacent semi-passive RIS elements have stronger channel correlation with each other, we assume that the semi-passive elements are continuously distributed over the RIS to better estimate the CSI.

We consider that both the BS and the RIS are uniform planar arrays (UPA) [19]; then the channel between the BS-RIS can be expressed as

(1) $$G=\sum_{l_{1}=1}^{L}a_{l_{1}}a_{RIS}\left(\varphi_{R,l_{1}},\theta_{R,l_{1}}\right)a_{BS}^{H}\left(\varphi_{T,l_{1}},\theta_{T,l_{1}}\right),$$

where *L* is is the number of paths between the BS-RIS, $a_{l_{1}}$ is the complex gain of the $l_{1}$th path, $\varphi_{l_{1}}$ and $\varphi_{l_{2}}$ is the azimuth angle, $\theta_{l_{1}}$ and $\theta_{l_{2}}$ is the elevation angle, $a_{RIS}\left(\varphi_{R,l_{1}},\theta_{R,l_{1}}\right)$, and $a_{BS}^{H}\left(\varphi_{T,l_{1}},\theta_{T,l_{1}}\right)$ is the antenna array response of the base station and RIS. The specific expressions can be expressed as

(2) $$\begin{array}{c} a_{RIS}\left(\varphi_{R,l_{1}},\theta_{R,l_{1}}\right)=\left[1,e^{j\frac{2\pi }{\lambda }d\;\sin\left(\varphi_{R,l_{1}}\right)\cos\left(\theta_{R,l_{1}}\right)},\ldots ,e^{j\frac{2\pi }{\lambda }dN_{1}\sin\left(\varphi_{R,l_{1}}\right)\cos\left(\theta_{R,l_{1}}\right)}\right]\\ ⊗\left[1,e^{j\frac{2\pi }{\lambda }d\;\sin\left(\varphi_{\left.R,l_{1}\right)}\right)},\ldots ,e^{j\frac{2\pi }{\lambda }dN_{2}\;\sin\left(\varphi_{\left.R,l_{1}\right)}\right)}\right], \end{array}$$

(3) $$\begin{array}{c} a_{BS}\left(\varphi_{T,l_{1}},\theta_{T,l_{1}}\right)=\left[1,e^{j\frac{2\pi }{\lambda }d\;\sin\left(\varphi_{T,l_{1}}\right)\cos\left(\theta_{T,l_{1}}\right)},\ldots ,e^{j\frac{2\pi }{\lambda }dM_{1}\sin\left(\varphi_{T,l_{1}}\right)\cos\left(\theta_{T,l_{1}}\right)}\right]\\ ⊗\left[1,e^{j\frac{2\pi }{\lambda }d\;\sin\left(\varphi_{T,l_{1}}\right)},\ldots ,e^{j\frac{2\pi }{\lambda }dM_{2}\;\sin\left(\varphi_{T,l_{1}}\right)}\right], \end{array}$$

where λ is the wavelength, $d=\frac{\lambda }{2}$ is the spacing between the antennas, $M=M_{1}\times M_{2}$, $N=N_{1}\times N_{2}$, and ⊗ is the Kronecker product. Similarly, we consider a uniform linear array (ULA) at the UE; the channel between the UE-RIS can be expressed as

(4) $$h_{r,k}=\sum_{l_{2,k}=1}^{L_{r,k}}\alpha_{l_{2,k}}a_{RIS}\left(\varphi_{R,l_{2,k}},\theta_{R,l_{2,k}}\right)\alpha_{t}^{H}\left(\phi_{T,l_{2,k}}\right),$$

where $L_{r,k}$ is is the number of paths between the UE-RIS, $\alpha_{l_{2}}$ is the complex gain of the $l_{2}$th path between the UE-RIS, $\varphi_{l_{1}}$ and $\varphi_{l_{2}}$ is the azimuth angle, $\theta_{l_{1}}$ and $\theta_{l_{2}}$ is the elevation angle, $a_{BS}\left(\varphi_{R,l_{1}},\theta_{R,l_{1}}\right)$, and $a_{RIS}^{H}\left(\varphi_{T,l_{1}},\theta_{T,l_{1}}\right)$ is the antenna array response of the base station and RIS. Similarily, the specific expressions can be expressed as

(5) $$a_{UE}\left(\phi_{T,l_{2,k}}\right)=\left[1,e^{j\frac{2\pi }{\lambda }d\;\sin\left(\phi_{T,L_{2,k}}\right)}\ldots e^{j\frac{2\pi }{\lambda }d\left(K-1\right)\;\sin\left(\phi_{T,l_{2,k}}\right)}\right],$$

(6) $$\begin{array}{c} a_{RIS}\left(\varphi_{R,l_{2,k}},\theta_{R,l_{2,k}}\right)=\left[1,e^{j\frac{2\pi }{\lambda }dN_{1}\;\sin\left(\varphi_{R,l_{2,k}}\right)\cos\left(\theta_{R,l_{2,k}}\right)},\ldots ,e^{j\frac{2\pi }{\lambda }dN_{1}\;\sin\left(\varphi_{R,l_{2,k}}\right)\cos\left(\theta_{R,l_{2,k}}\right)}\right]\\ ⊗[1,e^{j\frac{2\pi }{\lambda }d\;\sin\left(\varphi_{R,l_{2,k}}\right)},\ldots ,e^{j\frac{2\pi }{\lambda }dN_{2}\;\sin\left(\varphi_{R,l_{2,k}}\right)}], \end{array}$$

where *K* is the number of users.

### 2.2. Channel Estimation Protocol

By exploiting the channel coherence time differences and semi-passive elements, we propose a two-stage channel estimation protocol in a semi-passive RIS architecture. Firstly, because the RIS and BS locations are relatively fixed and the channel change rate is relatively slow, the BS-RIS channel is quasi-static.Then the estimated BS-RIS channel can be estimated once in a long time. In addition, due to the mobility of the UE, the UE-RIS channel is time-varying. Then the UE-RIS needs to be estimated frequently in a short time. Secondly, because of the semi-passive elements, both the BS-RIS channel and the UE-RIS channel can be estimated by RIS, but the UE-RIS has a faster channel change speed compared to the BS-RIS. If the UE-RIS time-varying channel is estimated on RIS, then the semi-passive elements need to be turned on frequently to achieve the optimal channel estimation performance, but this will increase the burden of RIS. Therefore, we only estimate the quasi-static channel of the BS-RIS on RIS in the first stage, and the time-varying channel of the UE-RIS is estimated by the BS in the second stage. In general, estimating the frequency of the BS-RIS is much lower than estimating the frequency of the UE-RIS, which reduces the average pilot overhead from a long time scale. Compared to the cascaded channel estimation method that estimates MNK coefficients, the proposed channel estimation protocol can recover the BS-RIS and the UE-RIS channels by estimating only $N_{0}M+NK$ coefficients. Therefore, the required pilot overhead is further reduced.

**Figure 2:**
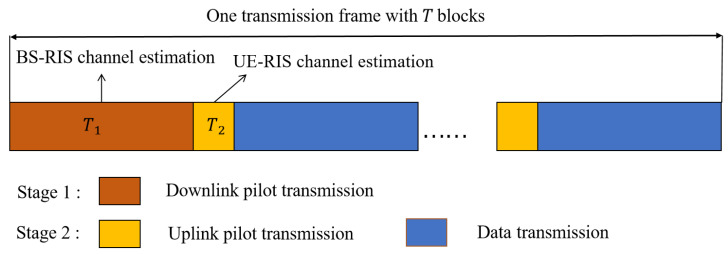
Channel estimation protocol.

As shown in Figure 2. There are *T* sub-frames during the signal transmission, where the first $T_{1}\ll T$ sub-frames estimates the BS-RIS channel and the second $T_{2}$ sub-frames are used to estimate the time-varying channel of the UE-RIS, and the channel is kept constant between each small time block. Specifically, in the $T_{1}$ sub-frames of the first stage, we consider the downlink communication, the BS transmits the pilot signal to the semi-passive RIS, the passive element turns off, the semi-passive element turns on and receives the pilot signal, and feeds the BS-RIS channel estimation result to the BS. In the $T_{2}$ sub-frames of the second stage, the BS estimates the UE-RIS channel by the uplink pilot signal sent by the UE. After the channel estimation is completed, the semi-passive element turns off the sensing mode and is used to reflect the signal together with the passive element during the data transmission. Based on this transmission protocol, we will discuss how to effectively estimate the BS-RIS channel versus the UE-RIS channel in the next two sections.

## 3. Stage 1: Estimation of the BS-RIS Channel

When the RIS is equipped with a small number of semi-passive elements, the CS algorithm can recover the complete channel of the BS-RIS using channel sparsity. However, this method usually requires solving a complex non-convex problem, it is difficult to obtain an optimal solution. In light of this, we propose an iterative re-weight-based channel estimation algorithm, which can estimate the BS-RIS channel with high accuracy by converting the non-convex problem into a new objective function by the iterative re-weight-based method and optimally solving it using the gradient descent method.

### 3.1. Downlink Pilot Transmission and Optimization Formulation

#### 3.1.1. Downlink Pilot Transmission

In the first stage, all the $N_{0}$ semi-passive elements operate in the sensing mode, while other passive components are off and do not participate in signal reflection. The BS sends pilot signal $x\in C^{M\times 1}$ to the RIS, then the signal is received at the semi-passive through a random combining matrix *W*. Thus, the received signal at the semi-passive after Q time slots is summarized as

(7) $$Y_{RIS}=W^{H}\bar{G}X+N,$$

(8) $$\bar{G}=\Gamma G,$$

where $Y_{RIS}=\left[y_{1},y_{2},\ldots y_{Q}\right]\in C^{N_{0}\times Q}$, $W\in C^{N_{0}\times N_{0}}$ is the random composite matrix, $X=\left[x_{1},x_{2},\ldots x_{Q}\right]\in C^{M\times Q}$, $\;\Gamma \in C^{N_{0}\times N}$ the selection matrix for selecting the semi-passive elements on the RIS, $\bar{G}\in C^{N_{0}\times M}$ is the channel between the BS and the RIS semi-passive elements, and $N\in C^{N_{0}\times Q}$ is the additive white Gaussian noise (AWGN) with $CN\left(0,\sigma_{n}^{2}\right)$.

As shown in Equation (1), to accurately estimate the BS-RIS channel, we need to estimate the complex gain of all paths $\alpha_{l_{1}}$, the departure angle $\alpha_{t}(\varphi_{T,l_{1}}$, $\theta_{T,l_{1}})$, and the arrival angle $a_{r}(\varphi_{R,l_{1}}$, $\theta_{R,l_{1}})$. In the framework of mmWave, the channel *G* of the BS-RIS can be transformed into

(9) $$G=A_{R}\left(\Phi \right)\mathrm{diag}\left(a\right)A_{T}^{H}\left(\Psi \right),$$

where $\Phi ={\left[\varphi_{R,1},\theta_{R,1},\varphi_{R,2},\theta_{R,2},\ldots ,\varphi_{R,L},\theta_{R,L}\right]}^{T}$, $\Psi ={\left[\varphi_{T,1},\theta_{T,1},\varphi_{T,2},\theta_{T,2},\ldots ,\varphi_{T,L},\theta_{T,L}\right]}^{T}$, $a={\left[a_{1},a_{2},\ldots ,a_{L}\right]}^{T}$, $A_{R}\left(\Phi \right)=\left[a_{RIS}\left(\varphi_{R,1},\theta_{R,1}\right),a_{RIS}\left(\varphi_{R,2},\theta_{R,2}\right),\ldots ,a_{RIS}\left(\varphi_{R,L},\theta_{R,L}\right)\right]$, $A_{T}\left(\Psi \right)=\left[a_{BS}\left(\varphi_{T,1},\theta_{T,1}\right),a_{BS}\left(\varphi_{T,2},\theta_{T,2}\right),\ldots ,a_{BS}\left(\varphi_{T,L},\theta_{T,L}\right)\right]$.

#### 3.1.2. Optimization Formulation

The above channel $\bar{G}$ estimation problem can be expressed as

(10) $$\min_{a.\Phi .\Psi }{∥a∥}_{0}\;s.t\;{∥Y_{RIS}-W_{t}^{H}\Gamma A_{R}aA_{T}^{H}X∥}_{F}\leq \varepsilon ,$$

where ε is a custom error threshold. Obviously, this is a sparse signal recovery problem for which the CS algorithm has good results, but its estimation accuracy is greatly affected by off-grid effects and the base mismatch problem, as well as the need to solve non-convex optimization problems with high computational complexity. To over-fit the data during the channel estimation process and cause inaccurate estimation, we add a regularization parameter and reformulate the above-obtained signal as

(11) $$\begin{array}{cc} \min_{a.\Phi .\Psi }L\left(a,\Phi ,\Psi \right) & ={∥a∥}_{0}\\ \; & +\xi {∥{\hat{Y}}_{RIS}-W_{t}^{H}\Gamma A_{R}aA_{T}^{H}X∥}_{F}^{2}, \end{array}$$

where ξ>0 is used to weigh the sparse constraint term against the error constraint term. Since the above problem is a non-convex optimization problem, it is difficult to find an optimal solution. Based on ref. [20], we use the logarithm and function instead of $L_{0}$ particularization; then we can obtain

(12) $$\begin{array}{cc} \min_{a.\Phi .\Psi }L\left(a,\Phi ,\Psi \right) & =\sum_{l=1}^{L}\log\left({\left|a_{l}\right|}^{2}+\delta \right)\\ \; & +\xi {∥{\hat{Y}}_{RIS}-W_{t}^{H}\Gamma A_{R}aA_{T}^{H}X∥}_{F}^{2}, \end{array}$$

where δ>0 is to ensure that the weight is not 0. Furthermore, solving the above problem remains a non-convex problem, and we use an iterative surrogate function instead of a logarithmic function. Then the minimization problem of $L\left(z,\theta_{R},\theta_{T}\right)$ is equivalent to the iterative optimization problem for the surrogate function.

(13) $$\begin{array}{cc} \min_{a.\Phi .\Psi }F\left(a,\Phi ,\Psi \right) & =\xi^{-1}a^{H}\Lambda^{\left(i\right)}a\\ \; & +{∥Y_{RIS}-W_{t}^{H}\Gamma A_{R}aA_{T}^{H}X∥}_{F}^{2}, \end{array}$$

where

(14) $$\Lambda^{\left(i\right)}=\mathrm{diag}\left(\frac{1}{\left|{\hat{a}}_{1}\left(i\right)\right|+\delta }\frac{1}{\left|{\hat{a}}_{2}\left(i\right)\right|+\delta }\cdots \frac{1}{\left|{\hat{a}}_{L}\left(i\right)\right|+\delta }\right),$$

where $\hat{a}\left(i\right)$ is the value of the value of the *i*th iteration. To obtain the optimal $F\left(a,\theta_{R},\theta_{T}\right)$, Equation (16) can be rewritten as

(15) $$\begin{array}{cc} F\left(a,\Phi ,\Psi \right) & =\xi^{-1}a^{H}\Lambda^{\left(i\right)}a+\sum_{p=1}^{Q}{∥y_{p}-W_{t}^{H}\Gamma A_{R}aA_{T}^{H}X∥}_{2}^{2}\\ \; & =\xi^{-1}z^{H}\Lambda^{\left(i\right)}a+\sum_{p=1}^{Q}{\left(y_{p}-\Xi_{p}a\right)}^{H}\left(y_{p}-\Xi_{p}a\right)\\ \; & =a^{H}\left(\xi^{-1}\Lambda^{\left(i\right)}+\sum_{p=1}^{Q}\Xi_{p}^{H}\Xi_{p}\right)a\\ \; & -a^{H}\left(\sum_{p=1}^{Q}\Xi_{p}^{H}y_{p}\right)-\left(\sum_{p=1}^{Q}y_{p}^{H}\Xi_{p}\right)a\\ \; & +\sum_{p=1}^{Q}y_{p}^{H}y_{p}, \end{array}$$

where $\Xi_{p}=W_{t}^{H}\Gamma A_{R}\mathrm{diag}\left(A_{T}^{H}x_{p}\right)$. Then taking the partial derivative of this gives

(16) $$\begin{array}{cc} \frac{\partial F\left(a,\Phi ,\Psi \right)}{\partial a}= & a^{H}\left(\xi^{-1}\Lambda^{\left(i\right)}+\sum_{p=1}^{Q}\Xi_{p}^{H}\Xi_{p}\right)\\ \; & -\left(\sum_{p=1}^{Q}y_{p}^{H}\Xi_{p}\right), \end{array}$$

By setting the derivative to zero, the minimum point *a* and the corresponding minimum value of $a_{\mathrm{opt}\;}\left(\Phi ,\Psi \right)$ can be obtained as

(17) $$a_{opt}^{\left(i\right)}\left(\Phi ,\Psi \right)=\left(\xi^{-1}\Lambda^{\left(i\right)}+\sum_{p=1}^{Q}\Xi_{p}^{H}\Xi_{p}\right)\left(\sum_{p=1}^{Q}\Xi_{p}^{H}y_{p}\right),$$

Substituting this into the original Equation (15) yields $F_{opt}\left(\theta_{R},\theta_{T}\right)$ the optimal value of

(18) $$\begin{array}{cc} F_{\mathrm{opt}\;}^{\left(i\right)}\left(\Phi ,\Psi \right)= & -{\left(\sum_{p=1}^{Q}\Xi_{p}^{H}y_{p}\right)}^{H}{\left(\xi^{-1}\Lambda^{\left(i\right)}+\sum_{p=1}^{Q}\Xi_{p}^{H}\Xi_{p}\right)}^{-1}\\ \; & \left(\sum_{p=1}^{Q}\Xi_{p}^{H}y_{p}\right)+\sum_{p=1}^{Q}y_{p}^{H}y_{p}, \end{array}$$

In this way, we obtain an estimation of the channel gain *a*. Then the channel estimation problem is further simplified to the estimation of parameters Φ and Ψ. The specific solving process will be described in the next section.

### 3.2. Propose Super-Resolution Channel Estimation Scheme

In the previous subsection, we transformed the BS-RIS channel estimation problem into a new optimization problem. Based on the optimization problem derived above, we propose an iterative re-weight-based channel estimation algorithm, as shown in steps 7 to 16 of Algorithm 1. In Equation (12), the former term constrains the sparsity of the estimation results, and the latter constrains the fitting error to avoid over-fitting. Specifically, a larger ξ can lead to more accurate solutions, while a smaller ξ will lead to over-sparse solutions and poor estimation results. Therefore, to have a good estimate for the iteration closer to the true value and obtain a smaller error, we update the regularization parameter in the iteration ξ. The updated equation is shown below

(19) $$\xi =\min\left(\frac{R}{r_{i}^{2}},\xi_{\max}\right),$$

where *R* is a custom scale factor, which is determined by factors such as signal-to-noise ratio and frequency interval. Here, $\xi_{\max}$ is used to limit the range of ξ to ensure that the algorithm is feasible, and $r_{i}$ is the residual from the previous step, i.e.,

(20) $$r_{i}={∥Y-W^{H}\Gamma A_{R}\left({\hat{\Phi }}^{i}\right)\mathrm{diag}\left({\hat{a}}_{i}\right)A_{R}\left(\Psi^{i}\right)X∥}_{F},$$

Moreover, to reduce the impact of quantization error on the channel estimation, we use the gradient descent method in machine learning to traverse the imaginary angle domain to obtain $\Phi_{R}^{\left(i+1\right)}$ that $\Psi_{R}^{\left(i+1\right)}$ which achieves super-resolution [21], with the following updated equation

(21) $$\Phi^{\left(i+1\right)}=\Phi^{\left(i\right)}-\eta \nabla_{\Phi_{R}}F_{opt}^{\left(i\right)}\left(\Phi^{\left(i\right)},\Psi^{\left(i\right)}\right),$$

(22) $$\Psi^{\left(i+1\right)}=\Psi^{\left(i\right)}-\eta \nabla_{\Psi_{R}}F_{opt}^{\left(i\right)}\left(\Phi^{\left(i\right)},\Psi^{\left(i\right)}\right),$$

where η is the step size to make sure $F_{\mathrm{opt}}^{\left(i\right)}\left(\Phi^{\left(i+1\right)},\Psi^{\left(i+1\right)}\right)\leq F_{\mathrm{opt}}^{\left(i\right)}\left(\Phi^{\left(i\right)},\Psi^{\left(i\right)}\right)$, and ∇ is gradient calculation operation. In addition, the number of reflected paths of mmWave are generally unknown in practical applications. During the iterative process of the proposed scheme, paths with too little gain will be considered as noise and pruned out, which makes the result more sparse, and the true path number is finally obtained by iterative pruning.

In the iterative update process, if randomly selected initial values cause significant computational complexity, then choosing the initial values for the iterations can reduce the number of iterations and thus achieve a reduction in complexity, as discussed in the next subsection.

### 3.3. SVD Algorithm of Preconditioning

To make the calculation easier, we adopt the singular value decomposition (SVD) algorithm to pre-process the received signal [21], as shown in steps 1 to 6 in Algorithm 1, to find the angular domain grid of arrival/departure(AoA/AoD) that is closest to the true value. Compared with setting the initial value arbitrarily, the pre-process can significantly reduce the computational complexity. The SVD decomposition of the received signal yields

(23) $$Y_{RIS}=U\Sigma V^{H},$$

where $Y_{RIS}$ is the signal received by the semi-passive element, $\Sigma =\mathrm{diag}\left(\xi_{1},\xi_{2},\ldots ,\xi_{\min(N_{0},Q)}\right)$$\in R^{N_{0}\times Q},U$ is the left singular matrix, V is the right singular matrix, and $U^{H}U=I_{N_{0}\times N_{0}}$, and $V^{H}V=I_{Q\times Q}$. According to Equation (10), it is obtained that

(24) $$Y_{RIS}=\left(W_{t}^{H}\Gamma A_{R}\right)a{\left(XA_{T}\right)}^{H}+N,$$

**Algorithm 1** Two-stage channel estimation algorithm Stage 1: Estimation of the BS-RIS Channel. Input: Receive signal $Y_{RIS}=\left[y_{1},y_{2},\ldots ,y_{Q}\right]$, combination matrix *W*, pilot signal $X=\left[X_{1},X_{2},\ldots X_{Q}\right]$, BS-RIS channel *G*, selection matrix Γ, trimming threshold $\Sigma_{t}$, termination thresholds ε and the number of paths $L_{init}$ to detect. Output: Estimated $A\left(\Phi \right)$ with $A\left(\Psi \right)$ and the path gain for each path.
1. $Y_{RIS}=U\Sigma V^{H}$2. Take the first $L_{init}$ columns of *U*, *V* and $L_{init}$ largest singular values.3. for $i=1,2,3,\ldots ,N_{it}$ do4. Calculated from Equations (26) and  (27).5. end for6. Output ${\hat{\varphi }}_{R}^{\left(0\right)}$, ${\hat{\theta }}_{R}^{\left(0\right)}$ and ${\hat{\varphi }}_{T}^{\left(0\right)}$, ${\hat{\theta }}_{T}^{\left(0\right)}$.7. Initialize $a_{\mathrm{opt}\;}$ according to Equation (17).8. Repeat:9. Update according to Equation (19) ξ.10. Calculated from Equation (18) $F_{\mathrm{opt}\;}\left(\Phi ,\Psi \right)$.11. Iterate according to Equations (21) and  (22) to find the optimal ${\hat{\varphi }}_{R}^{\left(i\right)}$, ${\hat{\theta }}_{R}^{\left(i\right)}$ and ${\hat{\varphi }}_{T}^{\left(i\right)}$, ${\hat{\theta }}_{T}^{\left(i\right)}$.12. Calculate the path gain according to Equation (17).13. Trim path number *l* if $a_{l}^{\left(i+1\right)}<\Sigma_{t}$.14. until $L^{i}=L^{\left(i+1\right)}$ and ${∥a^{\left(i+1\right)}-a^{i}∥}_{2}<\varepsilon$15. $\Phi ={\hat{\Phi }}^{\left(\mathrm{last}\;\right)}$, $\Psi ={\hat{\Psi }}^{\left(\mathrm{last}\;\right)}$, $a=a^{\mathrm{last}\;}$.16. Reconstructed from Equation (9) $G_{es}$.Stage 2: Estimation of the UE-RIS Channel.Input: Receive signal $Y_{k}=\left[Y_{1},Y_{2},\ldots Y_{T}\right]$, combination matrix *W*, pilot signal $X=\left[X_{1},X_{2},\ldots X_{T}\right]$, the BS-RIS estimated channel $G_{es}$, and the UE-RIS channel $h_{r,k}$.Output: $h_{r_{e}s}$.17. The PARAFAC decomposition channel problem is obtained according to Equation (34).18.Estimate $h_{r,es}$ according to Equation (35).


In SVD decomposition, the size of the singular value is proportional to the amount of information it bears, i.e., the larger the singular value, the closer the association with the true information. Furthermore, when the noise is small, the maximum L singular values are approximated by the number of paths, such that when i = 1, 2, ⋯L, we have

(25) $$\begin{array}{c} \left\{\begin{array}{c} u_{i}\approx W^{H}\Gamma a_{R}\left(\varphi_{R,l_{i}},\theta_{R,l_{i}}\right)/{∥W^{H}\Gamma a_{R}\left(\varphi_{R,l_{i}},\theta_{R,l_{i}}\right)∥}_{2}\\ \xi_{i}\approx |a_{l_{i}}|{∥W^{H}\Gamma a_{R}\left(\varphi_{R,l_{i}},\theta_{R,l_{i}}\right)∥}_{2}{∥X^{H}a_{T}\left(\varphi_{R,l_{i}},\theta_{R,l_{i}}\right)∥}_{2}\\ v_{i}\approx X^{H}a_{R}\left(\varphi_{R,l_{i}},\theta_{R,l_{i}}\right)/{∥X^{H}\Gamma a_{R}\left(\varphi_{R,l_{i}},\theta_{R,l_{i}}\right)∥}_{2}, \end{array}\right. \end{array}$$

where $u_{i}$ and $v_{i}$ are the i-th column of the left singular matrix *U* and the right singular matrix *V*, respectively. At this point, a crude estimate of the AOD and AOA can be obtained for

(26) $$A_{R}\left({\hat{\varphi }}_{R,l_{i}}^{\left(0\right)},{\hat{\theta }}_{R,l_{i}}^{\left(0\right)}\right)=\underset{\left(\varphi_{R},\theta_{R}\right)\in \Omega_{1}}{\arg\;\max}\;u_{i}W^{H}\Gamma A_{R}\left(\varphi_{R,l_{i}},\theta_{R,l_{i}}\right),$$

(27) $$A_{T}\left({\hat{\varphi }}_{T,l_{i}}^{\left(0\right)},{\hat{\theta }}_{T,l_{i}}^{\left(0\right)}\right)=\underset{\left(\theta_{T},\theta_{T}\right)\in \Omega_{2}}{\arg\;\max}\;v_{i}X^{H}A_{T}\left(\varphi_{T,l_{i}},\theta_{T,l_{i}}\right),$$

where $\Omega_{1}=\left\{\left(\frac{i}{N_{3}},\frac{j}{N_{4}}\right)|i=1,\ldots ,N_{3}-1;j=1,\ldots ,N_{4}-1\right\}$,$N_{3}\times N_{4}=N_{0}$. Similarly, $\Omega_{2}=\left\{\left(\frac{i}{M_{1}},\frac{j}{M_{2}}\right)|i=1,\ldots ,M_{1}-1;j=1,\ldots ,M_{2}-1\right\}$, $M_{1}\times M_{2}=M$.

By the SVD method, a coarse estimate around the exact value is obtained, and using it as the initial value of the iteration can move the estimate to the vicinity of the true value faster, thus reducing the computational complexity.

### 3.4. Pilot Overhead and Computational Complexity

#### 3.4.1. Pilot Overhead

In the first stage of estimating the BS-RIS channel, we use $N_{0}$ semi-passive elements to recover the MN coefficients of the BS-RIS, then RIS obtains at $N_{0}Q$ measurements for each sub-frame. We need at least $\left[\frac{MN}{N_{0}Q}\right]$ sub-frames. The minimum pilot overhead for the first stage is $\tau_{1}=T_{1}=Q\left[\frac{MN}{N_{0}Q}\right]$.

#### 3.4.2. Computational Complexity

In the first stage of the BS-RIS channel estimation, the algorithmic complexity of the super-resolution algorithm for solving the optimization problem is $O\left(MT\left(M+N_{0}\right)N_{0}^{2}M^{2}\right)$, and the complexity is further reduced by the SVD algorithm to $O\left(MT\left(M+N_{0}\right)N_{0}^{2}L^{2}\right)$.

## 4. Stage 2: Estimation of the UE-RIS Channel

In this section, based on the BS-RIS channels estimated in the previous stage $G_{es}$, we propose a channel estimation algorithm based on PARAFAC decomposition for estimating the UE-RIS channel ${\hat{h}}_{r,es}$.

### 4.1. Uplink Pilot Transmission and Problem Formulation

#### 4.1.1. Uplink Pilot Transmission

In the second stage, we consider an uplink transmission where the user transmits signals, and the semi-passive elements turn off the received signal mode and engage with the passive elements to reflect the signals, reflecting the user-transmitted pilot signals to the BS. To reduce the impact of inter-signal interference on the estimation performance, we use orthogonal pilot $x_{k}\in C^{K\times 1}$ to perform channel estimation, where the orthogonal pilot satisfies

(28) $$x_{k_{1}}x_{k2}^{H}=\left\{\begin{array}{cc} P_{MS}, & \mathrm{if}\;k_{1}=k_{2}\\ 0, & \mathrm{otherwise}, \end{array}\right.$$

where $P_{MS}$ is the transmit power of the UE; ${\left(.\right)}^{H}$ is the Matrix conjugate transpose. The uplink pilot transmission frame consists of *t* sub-frames, and each sub-frame lasts for *K* time slots. In the *t*-th sub-frame, the reflection coefficient vector at RIS is $\Theta \in C^{N\times 1}$. We keep the RIS phase shift constant for *K* time slots in each sub-frame. In one sub-frame, the signal received by the BS is modeled as

(29) $$y_{k}=W_{1}^{H}G_{es}diag\left(\Theta \left(1\right)\right)h_{r,k}x_{k}+n_{k},k=1,2\ldots ,K,$$

where $W\in C^{M\times M}$ is the composite matrix at the BS, $h_{r,k}\in C^{N\times K}$ is the first *k* UE-RIS equivalent channel of the UE-RIS, $G_{es}\in C^{N\times M}$ is the BS-RIS channel estimated in the first stage, $n_{k}$ is AWGN with $CN\left(0,\sigma_{n}^{2}\right)$, and $\Theta \left(1\right)={\left[\beta_{1}e^{j\theta_{1}\left(1\right)},\ldots ,\beta_{n}e^{j\theta_{n}\left(1\right)},\ldots ,\beta_{N}e^{j\theta_{N}\left(1\right)}\right]}^{T}$$\in C^{N\times 1}$ is a diagonal matrix of the reflection coefficients of the RIS with phase shift, the $\beta_{n}\in \left[0,1\right]\left(n=1,2,\ldots ,N\right)$ is the amplitude reflection coefficient, and $\theta_{n}\in \left[0,2\pi \right]\left(n=1,2,\ldots ,N\right)$ is the modulation phase. Then the signal received at *t* sub-frames is

(30) $${\bar{Y}}_{t}=W_{t}^{H}G_{es}diag\left(\Theta \left(t\right)\right){\left(Xh_{r,k}{}^{T}\right)}^{T}+N_{t,k},$$

where ${\bar{Y}}_{t}={\left[y_{k,1},y_{k,2},\ldots y_{k,t}\right]}^{T}$,$X={\left[x_{k,1},x_{k,2},\ldots x_{k,t}\right]}^{T}$, and $N_{k}={\left[n_{k,1},n_{k,2},\ldots n_{k,t}\right]}^{T}$. We consider Θ for a DFT matrix satisfying $\Theta *\Theta^{H}=tI$ in this paper.

#### 4.1.2. Problem Formulation

The above problem can be translated into an optimization problem

(31) $${\hat{h}}_{r,es}=\underset{h_{r,k}}{\mathrm{argmin}}{∥{\bar{Y}}_{t}-W_{t}^{H}G_{es}\Theta Z^{T}∥}_{F}^{2},$$

where $Z=Xh_{r,k}{}^{T}$. Since the UE-RIS is a low-dimensional channel and the LS algorithm has good results in dealing with low-dimensional channels [22], then the solution of Equation (31) obtained by least squares is ${\hat{h}}_{r,\theta s}={\left(A^{H}A\right)}^{-1}A^{H}{\bar{Y}}_{k}$, where $A=W_{t}^{H}G_{es}$. However, the channel $G_{es}$ is a sparse channel in mmWave systems, i.e., $\mathrm{Rank}\left(G_{es}\right)=L<\min\left(M,N\right)$ and $\mathrm{Rank}\left(A^{H}A\right)<\min\left(M,N\right)$, then the matrix inverse does non-existent when using the LS algorithm for channel estimation, which leads to inaccurate estimation results. To solve this problem, we propose a least squares method based on PARAFAC decomposition to estimate the UE-RIS channel in the next subsection, as shown in steps 17 to 18 of Algorithm 1.

### 4.2. The LS Algorithm Based on PARAFAC Decomposition

**Figure 3:**
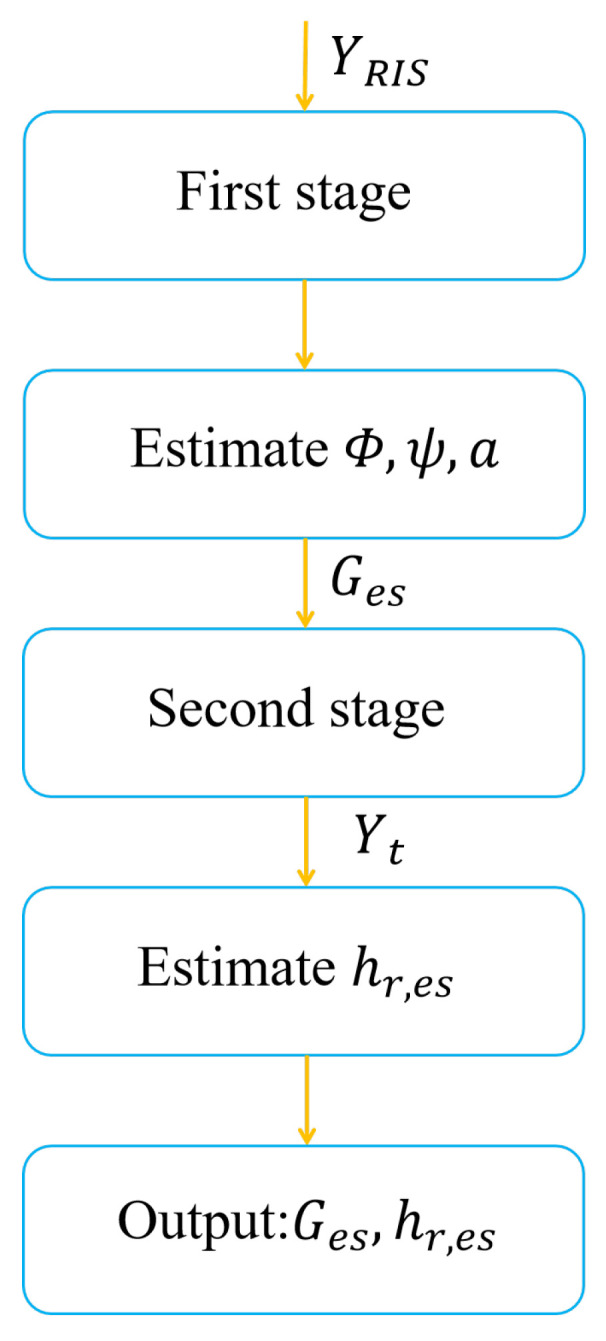
Two-stage channel estimation algorithm.

**Figure 4:**
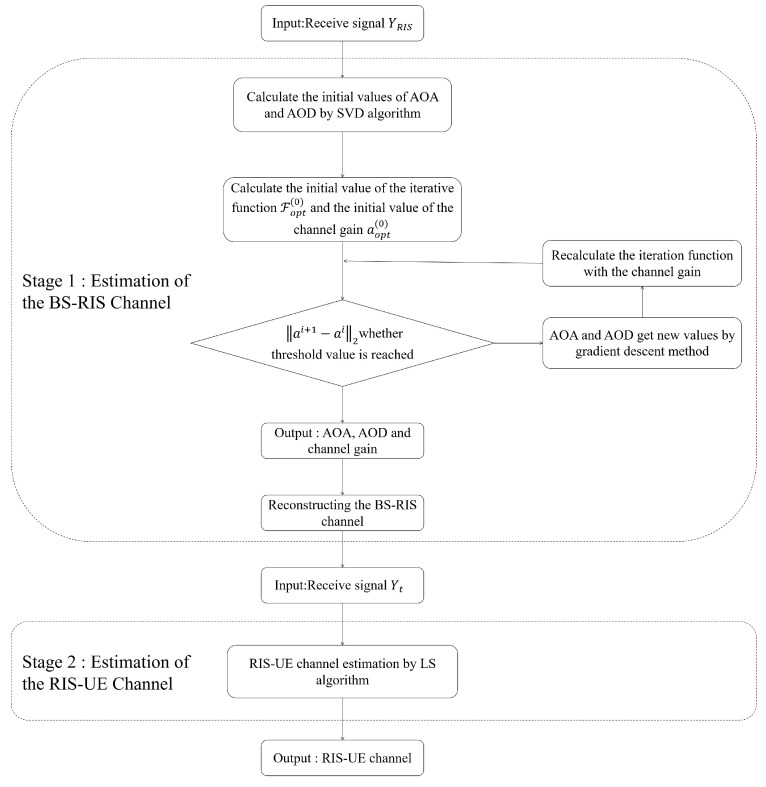
The actual process of the proposed two-stage channel estimation algorithm.

Based on the PARAFAC decomposition, the matrix ${\bar{Y}}_{k}$ can be considered as a three-way tensor Y∈T×M×K of the *k* the first positive matrix slice [23], then the noise-free received signal tensor can be obtained ${\bar{Y}}_{k}$ of the (ℓ,t,k) term

(32) $${\left[{\bar{Y}}_{t}\right]}_{\ell ,t,k}=\sum_{n=1}^{N}g_{\ell ,n}z_{t,n}s_{k,n},$$

where $g_{l,n}={\left[W_{t}^{H}G_{es}\right]}_{l,n}={\left[G\right]}_{l,n}$, $z_{t,n}={\left[Z\right]}_{t,n}$, $s_{k,n}={\left[S\right]}_{k,n}$. Exploiting the trilinearity of the PARAFAC decomposition, Equation (28) can be transformed as [24]

(33) $${\bar{Y}}_{t}=Xh_{r,k}{}^{T}{\left(\Theta ⋄\left(W_{t}^{H}G_{es}\right)\right)}^{T}+B_{t},$$

where ⋄ is the Khatri–Rao product, $B_{t}$ is the tensor unfolding of the noise. Since the $G_{\mathrm{es}}$ was estimated in the previous stage, solving the above problem can be translated into the following function

(34) $${\hat{h}}_{r,es}=\underset{h_{r,k}}{\mathrm{argmin}}{∥{\bar{Y}}_{t}-Xh_{r,k}{}^{T}{\left(\Theta ⋄\left(W_{t}^{H}G_{es}\right)\right)}^{T}∥}_{F}^{2},$$

where ${\hat{h}}_{r,es}$ is the estimated value of the UE-RIS channel. The solution is given by

(35) $${\hat{h}}_{r,es}=X^{†}{\bar{Y}}_{t}{\left[{\left(\Theta ⋄\left(W_{t}^{H}G_{es}\right)\right)}^{T}\right]}^{†},$$

where † is the pseudo-inverse of the matrix. To ensure the accuracy of the estimated solution, it is necessary to satisfy $rank\left(\Theta \right)+rank\left(W_{t}^{H}G_{es}\right)\geq N+1$. In other words, $t\geq N-L_{r,k}+1$. The detailed proof process is in the literature [24] and will not be overly described here. The proposed two-stage channel estimation algorithm is summarized in Figure 3 and Figure 4.

### 4.3. Pilot Overhead and Computational Complexity

#### 4.3.1. Pilot Overhead

In the second stage of estimating the time-varying channel UE-RIS, the RIS-UE channel has NK coefficients to be estimated. Since the BS is estimated in a sub-frame of *K* time slot to obtain MK coefficients, we need at least $N-L_{r,k}+1$ sub-frames. Therefore, the channel estimation for the second stage has a pilot overhead of $\tau_{2}=T_{2}=\left(N-L_{r,k}+1\right)K$.

#### 4.3.2. Computational Complexity

In the second stage of estimating the UE-RIS channel estimation, the algorithm complexity is determined by the LS channel estimation algorithm based on the PARAFAC decomposition, which is $O\left(MT\left(2M+1\right)+N^{2}\left(M+1\right)+\right.MN)$.

## 5. Simulation Results

In this section, we present simulation results to verify the effectiveness of the proposed channel estimation algorithm. In the simulations, we use normalized mean square error (NMSE) and average spectrum efficiency (ASE) to evaluate the channel estimation algorithm performance, whose expression is

(36) $$\mathrm{NMSE}\left(G_{es}\right)=10\log_{10}\left(\frac{1}{R}\sum_{r=1}^{R}\frac{∥G_{es}{-G∥}_{F}^{2}}{{∥G∥}_{F}^{2}}\right),$$

(37) $$\mathrm{NMSE}\left({\hat{h}}_{r,k}\right)=10\log_{10}\left(\frac{1}{R}\sum_{r=1}^{R}\sum_{k=1}^{K}\frac{{∥{\hat{h}}_{r,k}-h_{r,k}∥}_{F}^{2}}{{∥h_{r,k}∥}_{F}^{2}}\right),$$

(38) $$\begin{array}{c} \mathrm{NMSE}\left(\bar{H}\right)=10\log_{10}\left(\frac{1}{R}\sum_{r=1}^{R}\frac{∥\bar{H}{-H∥}_{F}^{2}}{{∥H∥}_{F}^{2}}\right), \end{array}$$

(39) $$ASE=E\left\{\log_{2}(det\left(1+{\left(N_{R}N_{T}\left(\sigma^{2}+NMSE\right)\right)}^{-1}HH^{H}\right))\right\},$$

where *R* is the number of Monte Carlo simulations and $\hat{G}$ is the result of the *r*-th the BS-RIS channel estimation, ${\hat{h}}_{r,k}$ is the result of the *r*-th UE-RIS channel estimation, $\bar{H}=\hat{G}\mathrm{diag}\left({\hat{h}}_{r,k}\right)$ is the *r*-th estimated cascaded channel, and $H=G\mathrm{diag}\left(h_{r,k}\right)$ is the true channel.

### 5.1. Parameter Setting and Simulation Analysis

The simulation parameters are set as follows: M=64, N=256, K=10, L=3, $L_{r,k}=6$, Q=64, the AoA and AoD parameters are uniformly generated from [−π/2,π/2], and the number of Monte Carlo simulations is 2000. The signal-to-noise ratio (SNR) is defined by $\mathrm{SNR}=\frac{1}{\sigma_{n}^{2}}$, where $\sigma_{n}^{2}$ is the noise variance. MATLAB R2019a is used for simulation. To verify the superiority of the proposed algorithm, we compare the following algorithms:

LS: The LS estimator from the literature [8] to estimate the channel.

MMSE: Based on the LS channel estimation algorithm, using the MMSE estimator from the literature Equation (10) to estimate the channel.

CS: Solving the non-convex optimization problem of Equation (12) using the CS algorithm which can be found in the literature [15].

Oracle LS: By assuming that the exact AOA and AOD are known to the RIS or BS and that the received signal is estimated to be a known common LS, the subspace is upper bounded with respect to the performance of the solution, which cannot realistically be achieved.

**Figure 5:**
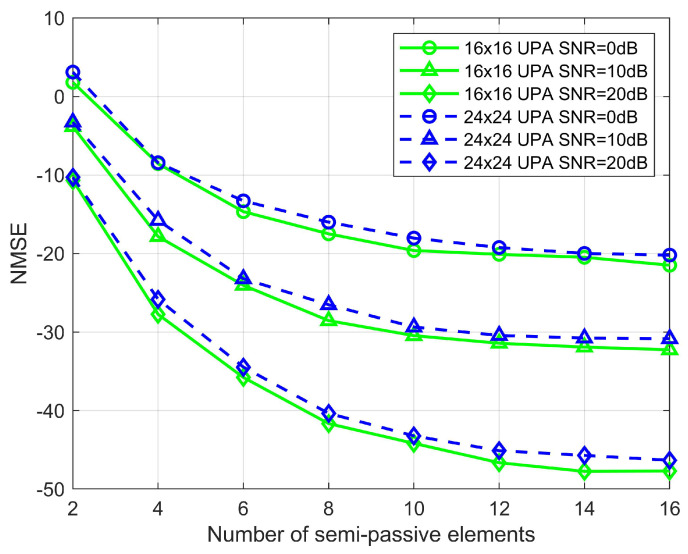
Effect of the number of semi-passive elements on NMSE, where M=64.

Figure 5 shows the number of semi-passive elements $N_{semi}=\sqrt{N_{0}}$ versus NMSE. We choose the UPAs with the sizes of 16×16 and 24×24 for comparison, and it can be seen that as the number of RIS elements increases, the estimation performance improves slightly, but the estimation performance decreases slightly as the number of RIS elements increases, which is due to the decrease in the proportion of semi-passive elements. It suggests that the number of semi-passive parts can be raised properly in actual applications to improve estimation performance.When the semi-passive elements are 64 and SNR=0,10,20 dB, the estimation accuracy of the proposed algorithm can reach $10^{-1.5}$, $10^{-3}$, and $10^{-4}$ orders of magnitude, which shows the superiority of the proposed algorithm in the channel estimation accuracy. Considering the trade-off between complexity and the performance affected by the number of semi-passive elements, we take the number of semi-passive elements to be 64 in the later simulations.

**Figure 6:**
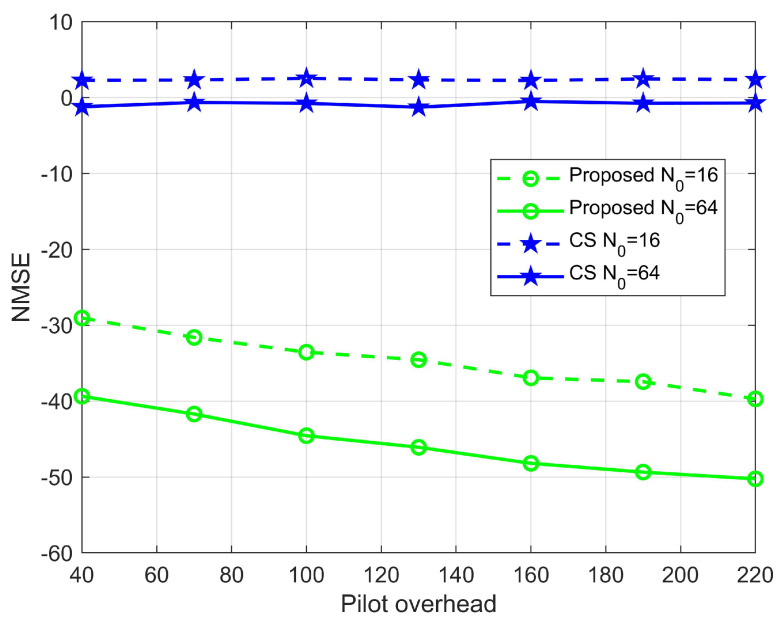
Influence of the pilot signal on the NMSE, where M=64, N=256.

Figure 6 shows the pilot overhead versus NMSE for the estimated BS-RIS channel with semi-passive elements of 16 and 64. We see that the scheme proposed in this paper performs better than the CS-based scheme for the same pilot overhead conditions. Moreover, the difference between the two grows as the training time increases. The CS-based method does not improve with increasing pilot overhead, mainly due to the limitation of the per-discrete AOD/AOA resolution. When the pilot overhead is 100, the estimation accuracy of the algorithm proposed in this paper can reach $10^{-3.5}$, $10^{-4.5}$ orders of magnitude; however, the estimation accuracy of CS is $10^{0}$, $10^{0}$ orders of magnitude, which shows the superiority of the algorithm proposed in this paper, which can still achieve a more desirable channel estimation with less pilot. Furthermore, the error of the scheme proposed in this paper gradually decreases as the number of semi-passive elements increases from 16 to 64, which is due to the increase in the proportion of semi-passive elements.

**Figure 7:**
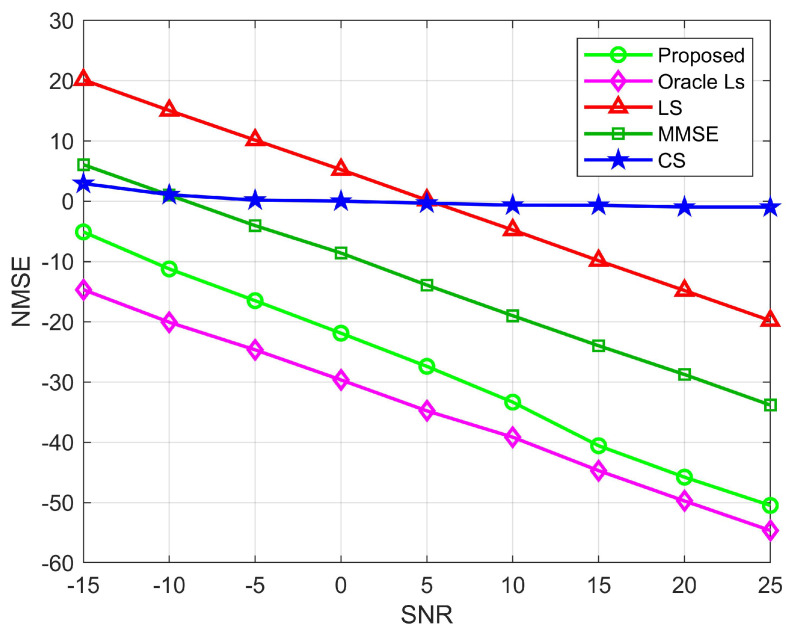
NMSE performance comparison for the BS-RIS channels, where M=64, N=256, $N_{0}=64$.

**Figure 8:**
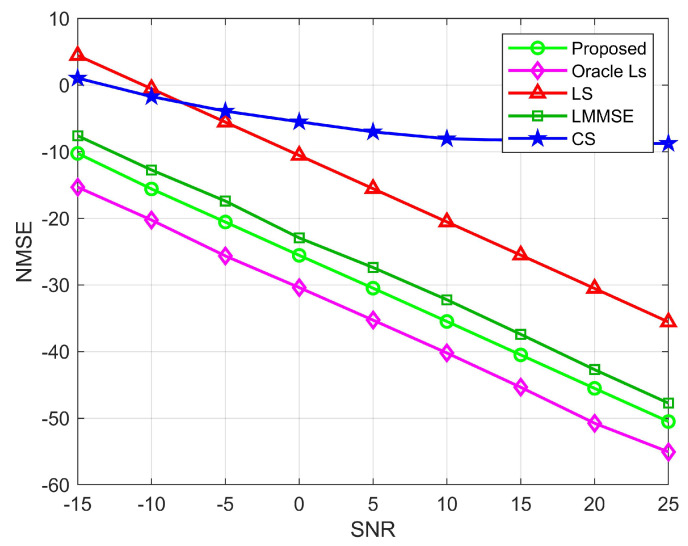
NMSE performance comparison for the UE-RIS channel, where M=64, N=256, K=10.

**Figure 9:**
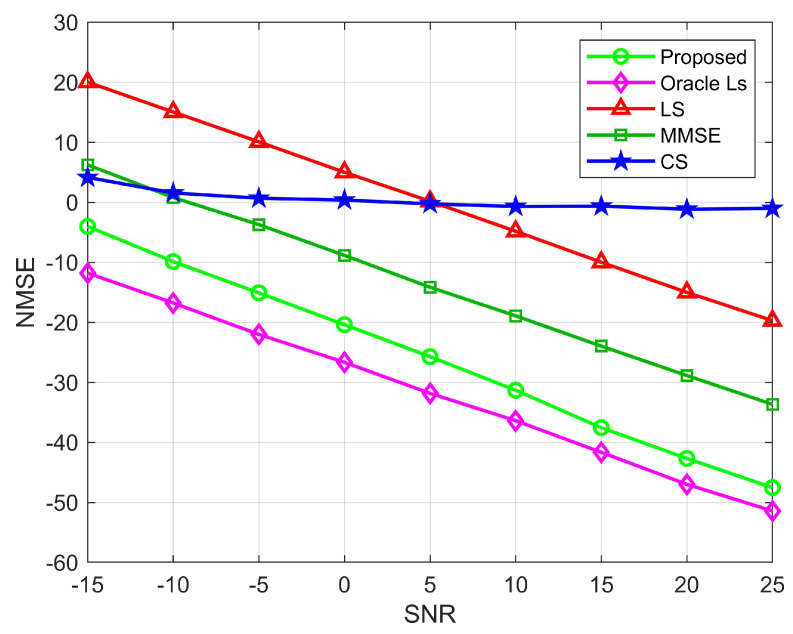
NMSE performance comparison for cascaded channels, where M=64, N=256, $N_{0}=64$, K=10.

Figure 7 and Figure 8 show the relationship between NMSE and signal-to-noise ratio for the BS-RIS and the UE-RIS channel estimation. We compare the NMSE of the proposed channel estimation with the LS, CS, and Oracle LS algorithms. Among them, the LS and the MMSE algorithms are carried out under the condition that all elements of the RIS are semi-passive elements. As can be seen from the figure, except for the CS algorithm, the rest of the algorithms decrease with the increase of SNR because the compressed sensing algorithm adopts a robust algorithm and has low sensitivity to noise. Because the real channel information is not necessarily in the preset dictionary, quantization errors are caused, resulting in poor estimation performance. The LS algorithm is sensitive to noise. Although the estimation decreases with the increase of SNR, the overall estimation performance is poor. When SNR=−15 dB, the proposed algorithm can reach the estimation accuracy of $10^{-0.5}$ and $10^{-1}$ orders of magnitude for the BS-RIS channel and the RIS-UE channel, respectively, which has obvious advantages over the LS, MMSE, and CS algorithms. When SNR=25 dB, the proposed algorithm can reach $10^{-5}$ orders of magnitude, respectively, and the estimation accuracy has obvious advantages over the other three algorithms. In general, the proposed algorithm has lower NMSE than the OMP, LS, and MMSE algorithms. However, the Oracle LS algorithm is more accurate for estimation because RIS uses the LS algorithm to estimate the perfect AOD and AOA. The algorithm is an ideal situation and has superior performance, but it cannot be applied in practice. Figure 9 shows the relationship between NMSE and SNR for cascaded channel estimation. It can be seen that for the cascaded channel, the estimation accuracy of the proposed division algorithm can reach $10^{-0.5}$, $10^{-5}$ orders of magnitude for SNR=−15 dB and SNR=25 dB, respectively, which are higher than those of the CS, LS, and MMSE algorithms for both high and low SNR cases, and is consistent with the results in Figure 6 and Figure 7. Therefore, we can see the superiority of the performance of the proposed algorithm.

**Figure 10:**
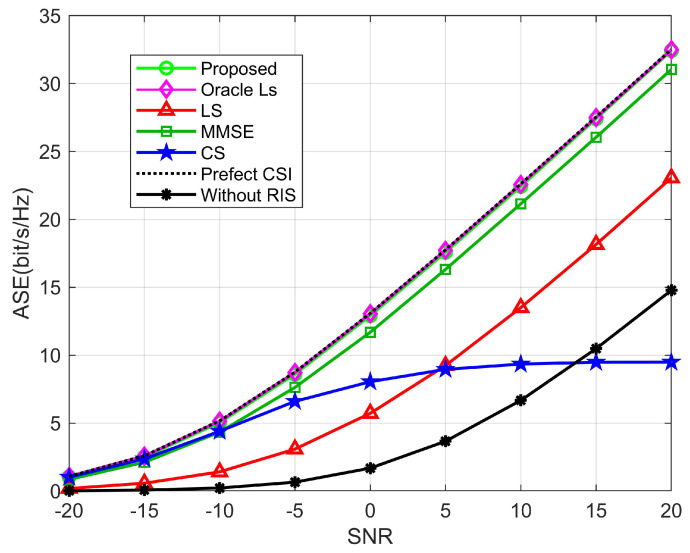
The ASE comparison with regard to SNR, where M=64, N=256, $N_{0}=64$, K=10.

Figure 10 shows the ASE of several methods for various SNRs, with the ideal CSI case serving as an upper bound. The proposed algorithm achieves a better spectral efficiency (SE) than other algorithms and is close to the ideal value. This is because the suggested channel estimation algorithm is better than other algorithms in terms of estimate accuracy, as shown in Figure 9. The CS algorithm grows slowly at SNRs of 5 to 10, which is caused by the stabilization of NMSE performance. When the SNR is 5, the performance of the proposed algorithm outperforms the CS, LS, and MMSE algorithms by 90.2%, 86.3%, and 10.5%, respectively. In addition, we present the SE performance without RIS, which verifies that the presence of RIS does improve the SE performance of mmWave communication because of the additional beamforming gain due to RIS. As a result, it can be demonstrated that the proposed scheme may successfully improve the SE. In addition, the quantified data when SNR = 10 dB are given in Table 2.

### 5.2. Pilot Overhead and Computational Complexity Analysis

#### 5.2.1. Pilot Overhead

**Figure 11:**
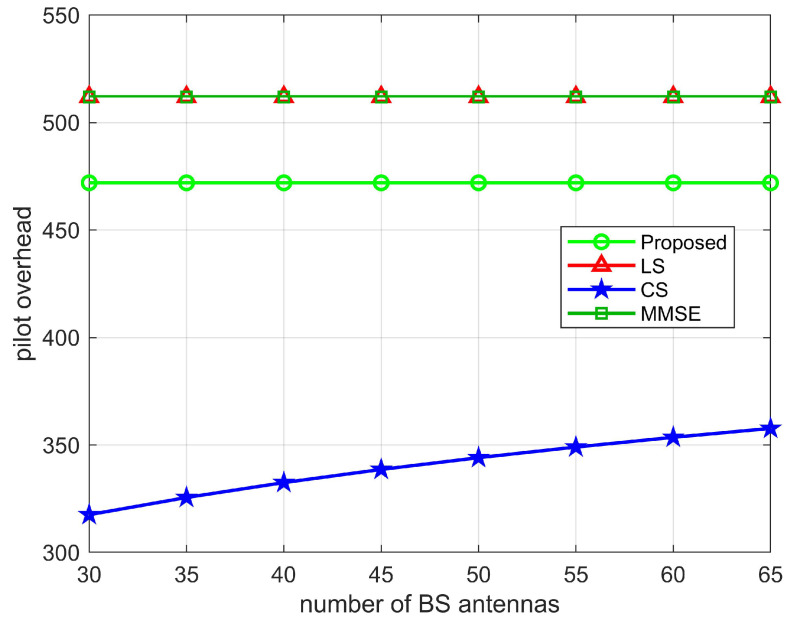
Pilot overhead versus number of antennas at BS, where N=64, K=10.

**Figure 12:**
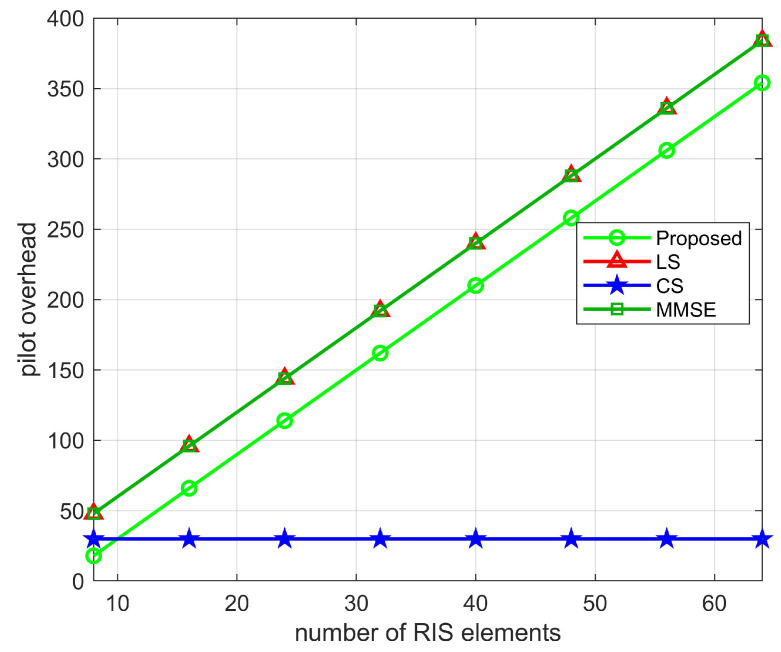
Pilot overhead versus the number of RIS elements, where M=64, K=10.

**Figure 13:**
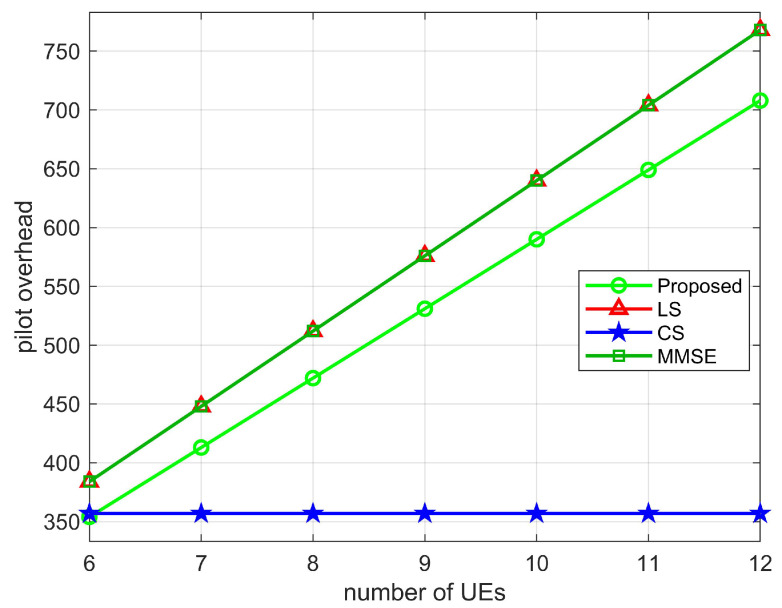
Pilot overhead versus number of UEs, where M=64, N=64.

To calculate the minimum pilot overhead, i.e., the average pilot overhead in the $T_{L}$ time period, the average pilot overhead is $\tau =\frac{T_{1}}{T_{L}}\tau_{1}=\frac{T_{1}}{T_{L}}M\left[\frac{N}{N_{0}}\right]$, due to $T_{L}>>T_{1}$, then $\frac{T_{1}}{T_{L}}\tau_{1}\approx 0$, the minimum pilot overhead of the proposed algorithm is $\tau +\tau_{2}=\left(N-L_{r,k}+1\right)K$. In Table 3, we compare the minimum pilot overhead of the proposed two-stage channel estimation with the LS, MMSE, and CS algorithms. As shown in Figure 11, Figure 12 and Figure 13, we plot the relationship between the pilot overhead and the number of BS antennas, the number of RIS elements, and the number of UEs. From the figures, we can see that the pilot overhead is lower than the LS and MMSE algorithms. Specifically, when the number of BS antennas is more than 30, the pilot overhead of the proposed algorithm is 472, which is lower than the LS algorithm and the MMSE algorithm. When the number of RIS elements as well as the number of UEs increases, the pilot overhead of the proposed algorithm, the LS algorithm, and the MMSE algorithm also increases, but the proposed pilot overhead is lower than these two algorithms. In addition, the CS algorithm uses the sparsity of the channel, and the pilot overhead is only related to the pre-defined grid matrix. Although the proposed algorithm has a pilot overhead higher than the CS algorithm, the proposed algorithm is more accurate than the CS algorithms in estimating. This also validates the superiority of the proposed algorithm.

#### 5.2.2. Computational Complexity

In the first stage of the BS-RIS channel estimation, the algorithmic complexity of the super-resolution algorithm for solving the optimization problem is $O\left(MT\left(M+N_{0}\right)N_{0}^{2}M^{2}\right)$, and the complexity is further reduced by the SVD algorithm to $O\left(MT\left(M+N_{0}\right)N_{0}^{2}L^{2}\right)$. In the second stage of estimating the UE-RIS channel estimation, the algorithmic complexity is determined by the LS channel estimation algorithm based on the PARAFAC decomposition with complexity $O\left(MT\left(2M+1\right)+N^{2}\left(M+1\right)+MN\right)$. In addition, we give the computational complexity of the compression-aware CS algorithm $O\left(2TN_{0}M\right)$; the computational complexity of the LS algorithm is $O\left(T{\left(NM\right)}^{2}\right)$.

## 6. Conclusions and Future Work

### 6.1. Conclusions

Aiming at the channel estimation problem of RIS-assisted MIMO systems, this paper proposes a two-stage channel estimation scheme. Firstly, we propose a two-stage channel estimation protocol to reduce the pilot overhead. The core idea of the protocol is to introduce semi-passive components to assist channel estimation and to use the coherent time difference between the BS-RIS channel and the RIS-UE channel to reduce the pilot overhead on a long time scale. Then, to estimate the BS-RIS channel accurately, we propose a super-resolution channel estimation algorithm. The algorithm moves the estimated AOA/AOD value to close to the real values through gradient descent and then obtains the accurate channel gain values based on the closed solution of the optimization problem. The algorithm avoids off-grid effects and power leakage and realizes the super-resolution estimation of the BS-RIS channels. Finally, to estimate the RIS-UE channel accurately, we propose an LS algorithm based on PARAFAC decomposition. The algorithm effectively utilizes the Khatri–Rao structure of the combined channel matrix to improve the estimation accuracy. Simulation results show that the proposed scheme can achieve higher accuracy than LS, CS, and MMSE schemes with less lead time overhead, and the spectral efficiency is improved by at least 10% compared to the other three algorithms. It also proves the superiority of the proposed scheme.

### 6.2. Future Work

The scheme proposed in this research can be utilized to greatly improve the performance of single RIS-assisted mmWave systems. We will look into the channel estimation challenge for multi-RIS-assisted mmWave systems in the future. Furthermore, in the entire application system, beamforming and RIS reflection coefficient design are critical. As a result, we will optimize the design of the beamforming vector and the RIS reflection coefficient matrix in tandem to improve system performance in the future study.

## Figures and Tables

**Table 1:** Summary of previous work.

RIS Configuration	Previous Work
Passive RIS	Channel estimation based on conventional least-squares (LS) algorithm for the RIS-assisted MIMO system [8,9].
	The MMSE algorithm is used to estimate the cascade channel for the RIS-assisted MIMO system [10].
	Channel estimation based on the methods of Lagrange multipliers and a dual ascent-based algorithm for the RIS-assisted MIMO system [11].
	Reference user-based channel estimation by using the common BS- RIS channel of the RIS-assisted MISO system [12].
	Channel estimation based on the methods of a sparse matrix decomposition and complementary channel estimation method for the RIS-assisted MIMO system [13,14].
	Compression-sensing-based channel estimation based on sparse re-presentation of cascaded channel [15].
Semi-passive RIS	Channel estimation based on compressed sensing for the RIS-assist-ed SISO system [16,17,18].

**Table 2:** The average spectrum efficiency of different algorithms when SNR = 5 dB.

Algorithm	Average Spectrum Efficiency (bps/Hz)
Perfect Channel	13.089
Proposed	12.911
LS	5.725
CS	8.053
MMSE	11.695

**Table 3:** Pilot overhead comparison of different channel estimation schemes.

Algorithm	Minimum Pilot Overhead
Proposed	$(N-L_{r,k}+1)K$
LS	KN
CS	$K[\frac{8LL_{r,k}-2}{M}]$
MMSE	KN

## Data Availability

Not applicable.

## References

[1] Nemati M., Park J., Choi J. (2020). RIS-Assisted Coverage Enhancement in Millimeter-Wave Cellular Networks. IEEE Access.

[2] Hassan K., Masarra M., Zwingelstein M., Dayoub I. (2020). Channel Estimation Techniques for Millimeter-Wave Communication Systems: Achievements and Challenges. IEEE Open J. Commun. Soc..

[3] Wu Q., Zhang S., Zheng B., You C., Zhang R. (2021). Intelligent Reflecting Surface-Aided Wireless Communications: A Tutorial. IEEE Trans. Commun..

[4] Wu Q., Zhang R. (2020). Towards Smart and Reconfigurable Environment: Intelligent Reflecting Surface Aided Wireless Network. IEEE Commun. Mag..

[5] Wu Q., Zhang R. (2019). Intelligent reflecting surface enhanced wireless network via joint active and passive beamforming. IEEE Trans. Commun..

[6] Hu X., Zhong C., Alouini M., Zhang Z. (2020). Robust design for IRS-aided communication systems with user location uncertainty. IEEE Commun. Lett..

[7] Mu X., Liu Y., Guo L., Lin J., Schober R. (2021). Joint Deployment and Multiple Access Design for Intelligent Reflecting Surface Assisted Networks. IEEE Trans. Commun..

[8] Mishra D., Johansson H. Channel estimation and low-complexity beamforming design for passive intelligent surface assisted MISO wire less energy transfer. Proceedings of the 2019 IEEE International Conference on Acoustics, Speech Signal Process (ICASSP’19).

[9] Jensen T.L., Carvalho E.D. On optimal channel estimation scheme for intelligent reflecting surfaces based on a minimum variance unbiased estimator. Proceedings of the IEEE International Conference on Acoustics, Speech and Signal Processing.

[10] Alwazani H., Kammoun A., Chaaban A. (2020). Intelligent reflecting surface-assisted multi-user MISO communication: Channel estimation and beamforming design. IEEE Open J. Commun. Soc..

[11] Lin J., Wang G., Fan R. (2020). Channel estimation for wireless communication systems assisted by large intelligent surfaces. arXiv.

[12] Wang Z., Liu L., Cui S. (2020). Channel estimation for intelligent reflecting surface assisted multiuser communications: Framework, algorithms, and analysis. IEEE Trans. Wireless Commun..

[13] He Z., Yuan X. (2020). Cascaded Channel Estimation for Large Intelligent Metasurface Assisted Massive MIMO. IEEE Commun. Lett..

[14] Ardah K., Gherekhloo S., de Almeida A.L.F., Haardt M. (2021). TRICE: A channel estimation framework for RIS-aided millimeter-wave MIMO systems. IEEE Signal Process. Lett..

[15] Wang P., Fang J., Duan H., Li H. (2020). Compressed channel estimation for intelligent reflecting surface-assisted millimeter wave systems. IEEE Signal Process. Lett..

[16] Taha A., Alrabeiah M., Alkhateeb A. (2021). Enabling large intelligent surfaces with compressive sensing and deep Learning. IEEE Access.

[17] Taha A., Alrabeiah M., Alkhateeb A. Deep learning for large intelligent surfaces in millimeter wave and massive MIMO systems. Proceedings of the 2019 IEEE Global Communications Conference (GLOBECOM).

[18] Liu S., Gao Z., Zhang J., Renzo M.D., Alouini M.-S. (2020). Deep denoising neural network assisted compressive channel estimation for mmWave intelligent reflecting surfaces. IEEE Trans. Veh. Technol..

[19] Zhu Z., Deng H., Xu F., Zhang W., Liu G., Zhang Y. (2022). Hybrid Precoding-Based Millimeter Wave Massive MIMO-NOMA Systems. Symmetry.

[20] Fang J., Wang F., Shen Y., Li H., Blum R.S. (2016). Super-resolution compressed sensing for line spectral estimation: An iterative reweighted approach. IEEE Trans. Signal Process..

[21] Hu C., Dai L., Mir T., Gao Z., Fang J. (2018). Super-resolution channel estimation for mmWave massive MIMO with hybrid precoding. IEEE Trans. Veh. Technol..

[22] Hu C., Dai L., Han S., Wang X. (2021). Two-Timescale Channel Estimation for Reconfigurable Intelligent Surface Aided Wireless Communications. IEEE Trans. Commun..

[23] Kolda T.G., Bader B.W. (2009). Tensor decompositions and applications. SIAM Rev..

[24] De Araújo G.T., De Almeida A.L.F., Boyer R. (2021). Channel Estimation for Intelligent Reflecting Surface Assisted MIMO Systems: A Tensor Modeling Approach. IEEE J. Sel. Top. Signal Process..

